# Flow Characteristics and Switching Mechanism of Bistable Slit Flow Actuated by Temperature

**DOI:** 10.3390/e25040650

**Published:** 2023-04-13

**Authors:** Huacheng Nie, Yuexia Lv, Tingting Du, Xinyu Song

**Affiliations:** 1 School of Mechanical Engineering, Qilu University of Technology (Shandong Academy of Sciences), Jinan 250353, China; 2 School of Energy and Power Engineering, Shandong University, Jinan 250061, China; 3 Shandong Institute of Mechanical Design and Research, Jinan 250031, China

**Keywords:** bistable slit flow, switching, flow regulation, temperature-driven, numerical simulation

## Abstract

The bistable flow is attractive as it can be analogous to a switch to realize flow control. Based on the previous studies on actuation technique, the present study first proposed temperature-driven switching of bistable slit flow. A two-dimensional numerical simulation was conducted to investigate the flow deflection characteristics and switching mechanism. It was concluded that the temperature gradient not only biases the slit flow but also locks it to the high-temperature side. The flow deflection angle became larger with the increase in temperature gradient. Being driven by the temperature, the flow can be switched from one side to the other. Furthermore, the fluid viscosity, which varies with temperature, determines the degree of flow deflection and the entire switching time. This research can enrich the active regulation of flow and has significant potential applications in thermal sensors, thermal detectors, microelectromechanical systems, biomedicine, and other equivalent fields.

## 1. Introduction

Bistable slit flow is a type of flow characterized by two stable equilibrium states: it can attach steadily to a wall based on the Coanda effect and can switch and reattach steadily to the other wall under any disturbance with enough driving energy. The bistable feature attracts tremendous attention because it can be analogous to a switch and applied as a valve or an oscillator for flow separation [1], cavity noise suppression [2], blunt-body drag reduction [3,4], and combustion control [5]. The actuation mechanism of the bistable mode plays an important role in flow regulation. The actuators range from passive actuation to active actuation. The major difference between these two types of actuation is whether there is an extra energy input or not. Mostly, the switch of bistable flow based on passive actuation [6,7,8,9] is realized by the design of a feedback loop, which transmits the pressure pulse to the flow-switching control. This benefits from having no moving parts and low energy consumption, but the oscillation frequency cannot be decoupled from the flow rate [10,11]. Comparatively, the active actuator controls the fluid by introducing signals, such as electric signal [12], acoustic signal [13], plasma signal [14], and piezoelectric control [15]. Most of them are applied at the outlet of a nozzle existing at low pressure. The flow is forced to switch to the other side by applying an input perturbation at the proper position. Some actuators are positioned on the jet centerline, such as cantilevered piezoelectric benders, whose oscillating ends point either upstream or downstream in the flow. Although additional energy is consumed, it still possesses the benefits of high accuracy and dependent switching control.

It is obvious that the switch to bistable flow requires sufficient potential energy input. Besides the actuators mentioned above, the temperature gradient also has the potential to trigger the flow due to its thermal potential energy. Research on temperature-driven switching of bistable flow was mostly presented in the flow passing two tubes for a certain pitch. When both cylinders are placed side by side, the wake normally presents a bistable phenomenon. Khalde et al. [16] carried out experiments on the characteristics of flow behavior around two circular cylinders and explored the influences of heat release on the near-wake structure. Their results demonstrated that the deflection of the gap flow was significantly affected by the heat release due to the low viscosity of water in the boundary layer over the cylinder. In the study of parallel microchannel reactors, Ma’moun et al. [17] found that temperature deviation in the barrier channels has a significant effect on flow distribution. This effect varies according to the relationship between viscosity and temperature.

Due to the limitations in visualization through experimental studies, a variety of researchers have carried out numerical simulation studies on the slit flow in different dimensions to investigate the mechanism of the temperature gradient on the flow field. Nikolay et al. [18] proposed a finite difference method to investigate the heat transfer and fluid flow over two rotating circular cylinders in a side-by-side arrangement at a low Re number. Jogee et al. [19] adopted a large-eddy simulation at *Re* = 3900 to reveal that convection and diffusion of heat happened mostly along the free shear layers. Based on a hybrid RANS-LES method, Lee et al. [20] concluded that the increase in temperature load enhanced the amplitude and frequency of shedding when the *Re* number was higher than 20,000. The study on the effect of a non-uniform temperature field shows the temperature gradient can affect the pressure distribution in the cross-section of triangular and trapezoidal pipe flows [21]. Additionally, in a plate, Takashi et al. [22] found that a small temperature difference can produce fluctuations in the velocity distribution for a variable fluid viscosity. Evidently, the above-mentioned research indicates that the temperature gradient has the ability to control flow. Since a large temperature gradient only exists in the thin boundary layer, it is difficult to impose a significant effect on the direction of the fluid in large-scale devices. However, the influence of thermal potential energy becomes remarkable when the device is scaled to a small size [23]. The numerical simulation results of the oscillator improved by Khalde et al. [24] show that it had a high heat and mass transfer effect in the small channel. Moreover, the control mechanism and flow separation mechanism of the surrounding flow and pipe flow are different. Therefore, for the slit flow, the mechanism of temperature trigger in micro-scale flow needs to be further explored.

Currently, many ways have been developed to drive bistable flow, but there are still few studies on temperature-driven bistable flow. The effect of thermal potential energy can affect the flow state and is expected to be applied in bistable flow. Based on the theory, the present paper utilized the temperature gradient as the trigger of bistable flow to investigate the flow characteristics and the switch mechanism based on the finite volume method (FVM). This research can enrich the actuation types and develop novel temperature-driven devices, such as thermal switches, thermal diodes, and thermal sensors.

## 2. Model and Method

### 2.1. Physical Model and Mechanism

**Figure 1:**
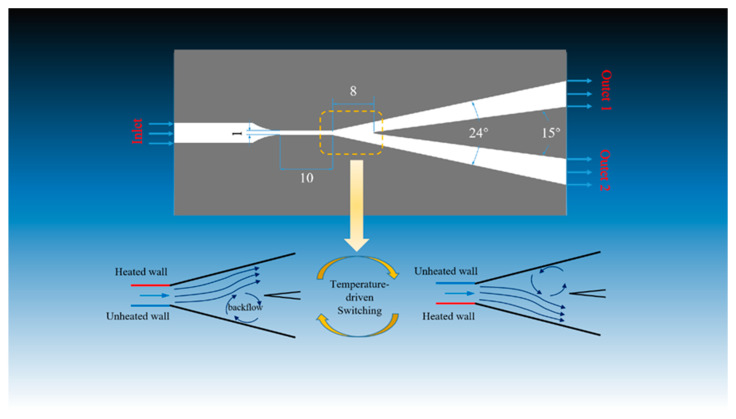
Physical model and schematic diagram of mechanism of bistable slit flow. The upper and lower walls of the 10 mm slit segment will be heated.

Mair et al. [13] innovatively proposed a geometric model to investigate the dynamics of acoustically driven bistable fluidic valves using a large-eddy simulation. In the present study, a temperature-driven geometric model is established by referring to the above-mentioned geometric model, especially by setting the outflow width at 1 mm. The mechanisms of different driving methods are quite different. Therefore, it is correspondingly in the interest of the present study to investigate the feasibility of the proposed geometric model for bistable flow via temperature control. Details of the structural parameters as well as the schematic diagram of the mechanism for temperature-driven switching of the bistable slit flow are given in Figure 1.

In this work, the fluid is introduced into the channel through the inlet, the length of which is set to ensure the flow is uniform before it enters the slit. The slit is 1 mm in width and 10 mm in length, which is calculated based on the boundary layer theory in order to obtain the largest angle of the flow deflection. One side of the slit is heated to a higher temperature, while the other side is kept at the same temperature as the fluid. The temperature gradient imposed between the two sides of the slit causes the fluid to deflect towards the side with a higher temperature. By reversing the temperature gradient vector, the flow deflection can be switched, enabling temperature control of bistable flow. Aiming to enable more fluid to follow the Coanda effect, the split with an angle of 15° is designed to be located 8 mm from the edge of the slit outlet, and the opening angle between the outlets is 24°. The flow through the two outlets is set to free flow.

### 2.2. Governing Equations

Considering that water density, thermal conductivity, and specific heat capacity are little affected by temperature and pressure in this simulation, they are all expressed in the form of constants in the governing equation. Therefore, for the viscous, incompressible fluid flowing in a slit, the governing equations of the computational domain are presented as follows:

Mass equation:

(1) $$\rho \frac{\partial u}{\partial x}+\rho \frac{\partial v}{\partial y}=0$$

Momentum equations:

(2) $$\rho \frac{\partial u}{\partial t}+\rho \frac{\partial }{\partial x}\left(u^{2}\right)+\rho \frac{\partial }{\partial y}\left(vu\right)=-\frac{\partial p}{\partial x}+\frac{\partial }{\partial x}\left(\mu \left(T\right)\frac{\partial u}{\partial x}\right)+\frac{\partial }{\partial y}\left(\mu \left(T\right)\frac{\partial u}{\partial y}\right)\;\rho \frac{\partial v}{\partial t}+\rho \frac{\partial }{\partial x}\left(uv\right)+\rho \frac{\partial }{\partial y}\left(v^{2}\right)=-\frac{\partial p}{\partial y}+\frac{\partial }{\partial x}\left(\mu \left(T\right)\frac{\partial v}{\partial x}\right)+\frac{\partial }{\partial y}\left(\mu \left(T\right)\frac{\partial v}{\partial y}\right)$$

Energy equation:

(3) $$\rho c_{p}\frac{\partial T}{\partial t}+\rho c_{p}\frac{\partial }{\partial x}\left(uT\right)+\rho c_{p}\frac{\partial }{\partial y}\left(vT\right)=\;\lambda \frac{\partial^{2}T}{\partial x^{2}}+\;\lambda \frac{\partial^{2}T}{\partial y^{2}}$$

where *u* and *v* are the velocity components in the *x* and *y* directions, respectively, m/s; *p* is the pressure, Pa; *ρ* is the fluid density, which keeps constant, kg/m^3^; *μ* is the fluid dynamic viscosity, which varies with the temperature, Pa·s; *c_p_* is the specific heat capacity of the fluid, J/(kg·K); *λ* is the thermal conductivity of the fluid, W/(m·K). *T* is the temperature of the fluid, K.

### 2.3. Meshing and Validation

**Figure 2:**
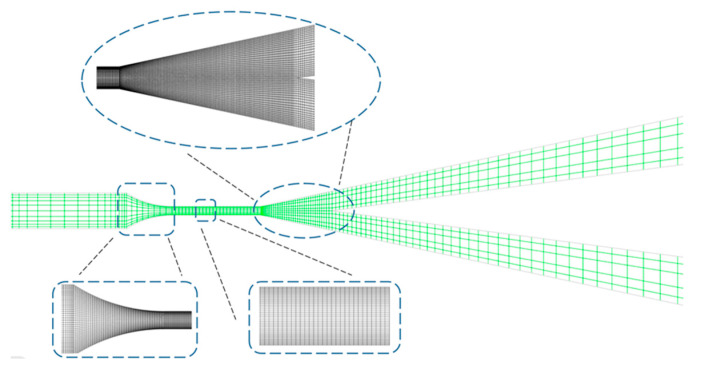
Meshes of the overall and local fluid domain. The slit inlet section, slit section, and slit outlet section are encrypted.

Taking into account the simple physical structure and the flow state in the slit, the present paper adopts the two-dimensional laminar model and FVM for numerical simulation. The structured quadrilateral mesh is employed due to its simplicity and efficiency in this scenario. Adaptive mesh refinement is used to present the details of flow and heat transfer in the boundary layers. The meshing of the overall and local fluid domains is shown in Figure 2. The grid independence is verified based on the method proposed by Peng et al. [25] by testing the outflow velocity in coarse, medium, and fine grids with the same inlet flow velocity. The results are shown in Table 1. The maximum deviation between the coarse and fine grids is 5.96%, showing that even the coarse mesh can meet the requirement of independence. Given the consumed time and the simulation accuracy, the medium grid is selected for the numerical simulation part.

The fluid is water and is assumed to be an incompressible fluid with a no-slip boundary and constant physical properties except viscosity. Considering that the viscosity change of water has a significant influence on the flowing boundary layer in the microchannel, the water viscosity variation with temperature is fitted by MATLAB based on the data from the literature [26], as shown in Equation (4). The average inlet flow velocity is 0.08 m/s with a constant temperature of 20 °C. The time step in the simulation is set at 0.02 s, and the number of maximum iterations is 50.

(4) $$\mu =1.932*10^{-7}T^{2}-0.000138T+0.0249$$

To characterize the thermal effects on the flow, the local Nusselt number (*Nu*(*x*)) is investigated, especially in the section of fully-developed thermal boundary layers [27,28]. The local Nusselt number (*Nu*(*x*)) is expressed in Equations (5)–(7), referring to research carried out by Su et al. [28] and Shah et al. [29]. The results are compared with the Nusselt number of the fluid in the microchannel described above.

(5) $$Nu\left(x\right)=\frac{h\left(x\right)\cdot D_{h}}{\lambda }$$

(6) $$h\left(x\right)=\frac{q_{x}}{T_{w}-T_{f}}\;$$

(7) $$T_{f}=\frac{\int_{0}^{d}uTdy}{\int_{0}^{d}udy}$$

According to the literature [28], define dimensionless x^*^:

(8) $$x^{*}=\frac{x}{D_{h}RePr}$$

Where *h*(*x*) is the wall convective heat transfer coefficient at position *x*, W/(m^2^·K); *q_x_* represents the local heat flux, W/m^2^; *T_w_* and *T_f_* are the wall temperature and the average fluid temperature read on the cross-section at position *x*, respectively, K; *D_h_* represents the characteristic length and is twice the slit width, m. *u* is the velocity of the fluid, m/s; and the Reynolds number and Prandtl number are expressed as *Re* and *Pr*, respectively.

**Figure 3:**
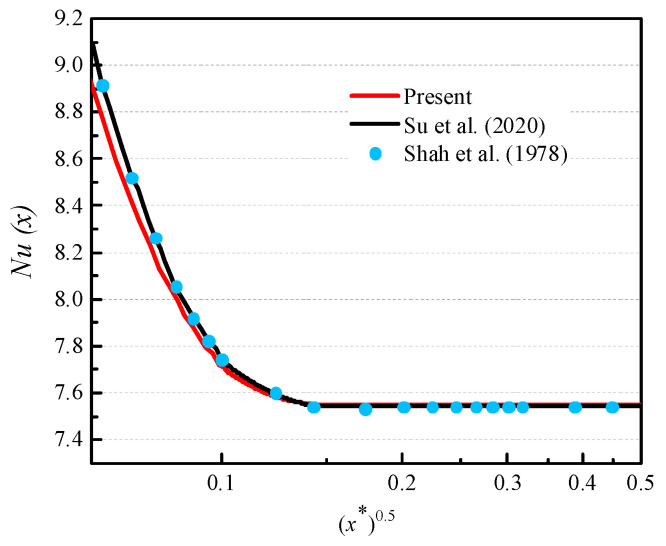
Variation of local Nusselt numbers with dimensionless axial distance for different research. The numerical results are almost identical to those in other literatures [28,29].

Figure 3 shows the variation relationship of *Nu*(*x*) with (x^*^)*^0.5^*. It can be evidently observed that the numerical calculation results agree well with the data given in the literature [28,29]. There is only a slight deviation in the inlet section, which is induced by the shrinkage inlet, with a change in the cross-section area where the fluid is accelerated. Therefore, Figure 3 proves that the proposed numerical method is reliable in characterizing flow and heat transfer of the fluid both in the slit and in the channels to the outlets.

## 3. Results and Discussion

### 3.1. Flow Characteristics in Slit Imposed by Heat

**Figure 4:**
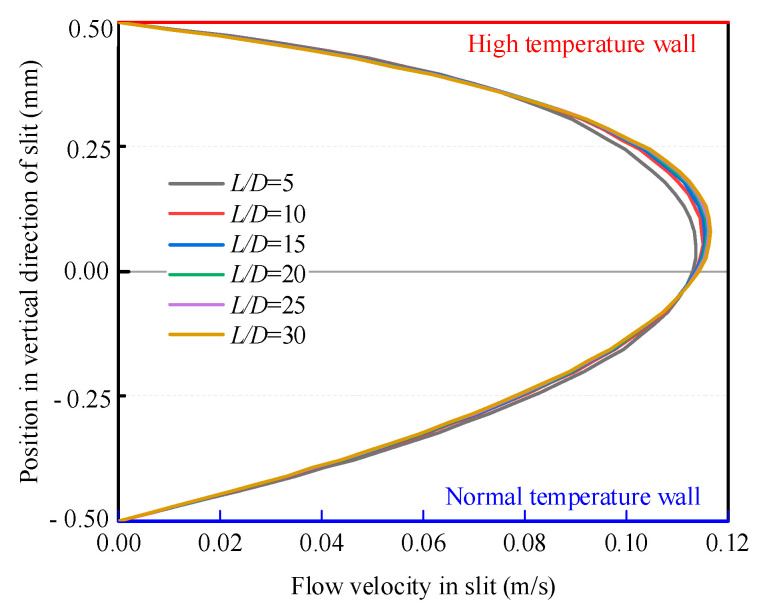
Velocity profile in different *L*/*D* ratios under the same temperature difference. The line for *L*/*D* = 5 is different from the other five.

It is well known that the velocity distribution of the viscous laminar flow between two stationary parallel plates shows the characteristic symmetry parabolic flow profile based on the theory of Poiseuille flow. But when one of the plates is heated, the parabolic flow shows an asymmetry feature. By using the asymmetry of the flow distribution in the slit, the flow can be disturbed and deflected under the Coanda effect. In order to investigate the influences of the heated length on the flow asymmetry, the velocity distribution in the slit is investigated at five different ratios of the slit length (*L*) to the slit width (*D*), which are *L*/*D* = 5, 10, 15, 20, 25, and 30. At a Reynolds number of 147, it can be seen in Figure 4 that the parabolic profile deviates from the balance to the side of the heated wall. Additionally, the asymmetry increases with the increase in the ratio, indicating that the temperature gradient can impact the flow asymmetry. The greater the heat input, the larger the asymmetry. In Figure 4, when *L*/*D* = 5, the position of the highest velocity deviates from the centerline (0 mm) by 0.054 mm. When *L*/*D* = 10, the deviation rises to 0.073 mm. After that, even if *L*/*D* is increased to 30, the deviation only rises to 0.083 mm, an increase of only 14% in deflection. Therefore, the heated length has less influence on the deflection when the ratio is higher than 10. The simulation adopted a heating model with a width-to-length ratio of 1:10. In this way, the deflection of the slit outlet can be nearly maximized.

### 3.2. Effect of Temperature Gradient on Bistable Slit Flow

**Figure 5:**
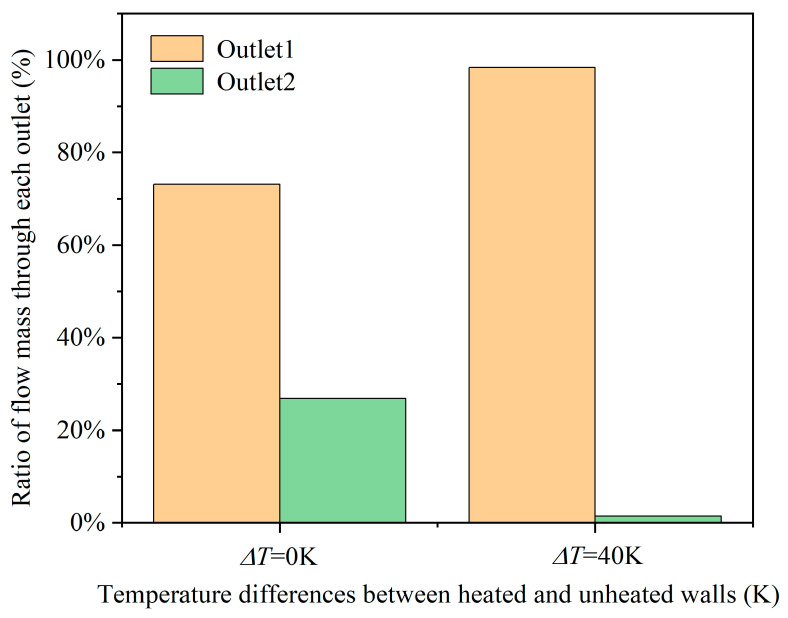
Ratio of flow mass through each outlet at different wall temperature differences. Wall heating allows more flow to exit through the thermal outlet.

The temperature gradient has a significant influence on the flow in the slit, which causes a large deflection of flow out of the slit. To further explore the effect of temperature gradient on the bistable slit flow, the flow characteristics at different temperature gradients are investigated. The slit flow velocity is kept at 0.08 m/s, and the temperature is kept constant at 20 °C. The Reynolds number is calculated based on the viscosity at the average outlet section temperature of the slit, taking into consideration that the flow is heated. The Reynolds number of this section is 167, indicating that all simulations are in the laminar flow regime. One wall is set to the same temperature as the fluid, while the other wall is heated in order to generate the temperature gradient. Figure 5 presents the change in flow behavior due to the existence of the temperature gradient. It is astonishing that the temperature gradient imposed on the slit can induce a significant difference in the mass flow rate through both outlets. The flow mass ratio between outlet1 and outlet2 is 2.72 when both walls are kept at the same temperature. This ratio is solely due to the Coanda effect, and the attached walls are random for symmetric models. However, this ratio jumps to 67.79 when the wall on the same side as outlet1 is heated to 60 °C. The fact is that most fluid flows along the heated side, demonstrating that the temperature gradient controls the flow deflection and aggravates the nonuniform distribution of the flow.

**Figure 6:**
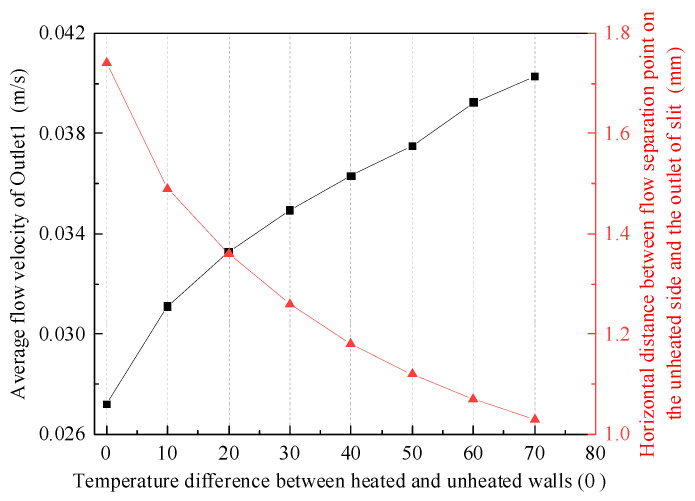
Variation of mean flow velocity at the outlet1 (black line) and movement of flow separation point towards the outlet of the slit in a horizontal direction (red line) at different temperature differences. With the increase in temperature difference, the flow separation on one side of the wall advances, while the flow attachment effect on the other side improves.

**Figure 7:**
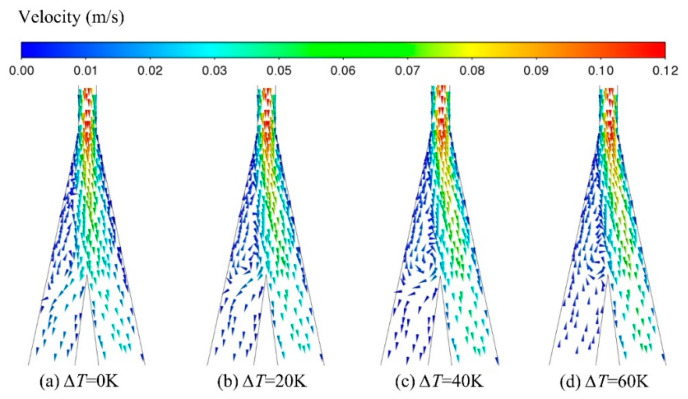
Velocity vector diagram of flow attached to the wall at different temperature differences. The backflow on the left side of the shunt increases with the increase in temperature gradient.

The effect of the temperature gradient on the flow characteristics was further investigated. The inlet flow temperature is kept at a constant value, and the temperature difference between the two walls is set as *∆T* = 0 K, 10 K, 20 K, 30 K, 40 K, 50 K, 60 K, and 70 K, respectively. It is observed from Figure 6 (black line) that the flow mass through outlet1 increases with the increase in the temperature gradient. Especially when the heating temperature reached 60 K and 70 K, the ratio of the flow rate on the heating side to that of the normal side reached 67.79 and 83.58, respectively. To gain insight into the effect on the flow deflection, the flow separation along the side of the low-temperature wall is investigated. In fluid dynamics, boundary layer separation occurs in flows where there is a decrease in velocity and an increase in pressure. In this study, the boundary layer detaches from the surface easily when the flow passes through the widening passage. That is the reason why there is a backflow along the wall, despite both walls being at the same temperature, as shown in Figure 7. According to the research results related to bistable flow [30], the deflection of fluid flowing out of a slit is random without any extra disturbance. However, the heat as an input disturbance can realize the control of the bistable state. When the heat is applied on one side, the deflection is locked to the heated wall, and the backflow along the other side gradually develops into a vortex. The local Reynolds number in the center area of the backflow gradually increases from 9.3 to 15.6. The vortex expands the streamline in the vertical direction, which pushes more fluid to flow by the heated passage side. Meanwhile, the flow vortex occurs closer to the outlet of the slit with an increase in temperature difference. Figure 6 (red line) presents the horizontal distance between the flow separation point and the slit outlet. The location of the separation point can be determined by the location where the shear stress of the wall is zero:

(9) $$\tau_{0}=\mu {\left(\frac{\partial u}{\partial y}\right)}_{0}$$

Due to the viscosity of the fluid, zero shear stress indicates zero velocity gradient at the wall. This demonstrates that when the temperature difference is increased to 70 K, the flow separation points to the outlet of the slit moving backward nearly 0.71 mm. Therefore, the flow separation is the critical factor for the flow deflection, while the temperature gradient determines the position of the flow detachment.

**Figure 8:**
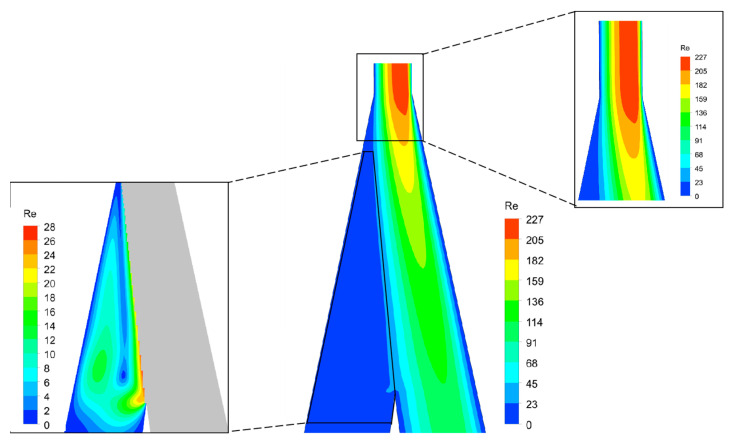
Local Reynolds number distribution at the temperature gradient of 40 K/mm. Partial amplification was carried out on the left side of the diversion port and the slit outlet, respectively.

In order to clearly characterize the specific conditions of fluid flow, Figure 8 shows the specific distribution of the local Reynolds number at a 40 K temperature difference. The characteristic length is still twice the slit width. Since the fluid is defined as incompressible, the Reynolds number is a function of flow rate and viscosity. As shown in the overall diagram, the local Reynolds number is basically determined by the flow rate. In addition, it can be seen from the local magnifying diagram at the outlet of the slit that the distribution of the local Reynolds number tends to favor the heating side before flowing out of the slit, indicating that the influence of viscosity on the fluid at this location cannot be ignored. To express the flow condition of the vortex at the normal temperature side, the local Reynolds number of this side is enlarged. The results show that the local Reynolds number of this side tends to be consistent with the vortex position in Figure 7c, and the maximum Reynolds number is 13.8. This indicates that reflux can promote flow separation.

### 3.3. Switching Characteristics of Temperature-Driven Bistable Flow

**Figure 9:**
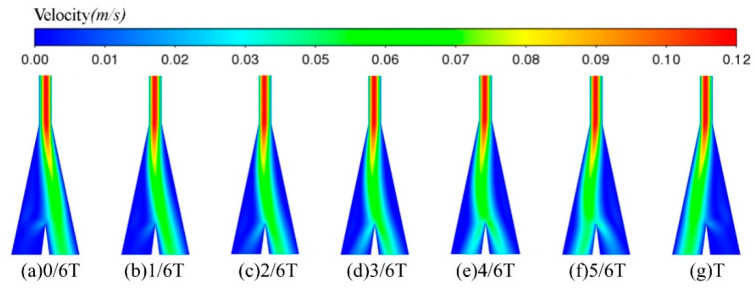
Process of bistable slit flow switching driven by temperature gradient (T represents the switching period, 0/6 T is the switching heating wall moment). The temperature gradient controls the flow switching.

To investigate the control of temperature gradient on the bistable flow, the flow behavior is recorded when the temperature gradient vector is suddenly reversed in direction, as shown in Figure 9. It can be observed that the main flow successively experiences a process of attachment to one side, detachment, and then reattachment to the other side. The whole switching process costs about 4.54 s. Although this period is longer than that of some actuators, such as acoustics [13], plasma [14], and other high-energy perturbations, it is still confirmed that a temperature gradient works as an effective trigger. Furthermore, it can also be predicted that the larger the temperature gradient, the shorter the consumption time. This can be attributed to the fact that more energy is introduced for the flow deflection because the momentum and kinetic energy are both enhanced by the temperature gradient.

### 3.4. Mechanism of Flow Deflection Driven by Temperature Gradient

**Figure 10:**
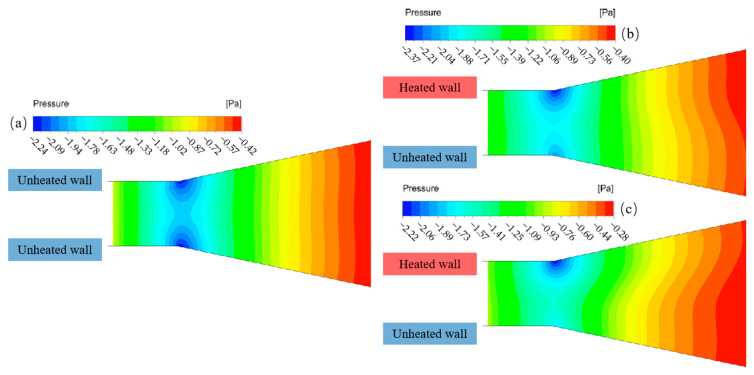
Pressure contour of flow field affected by temperature difference (*∆T*): (**a**) *∆T* = 0 at time of 0.2 s; (**b**) *∆T* = 40 K at time of 0.2 s; (**c**) *∆T* = 40 K at time of 4.12 s. The unilateral wall heating results in uneven pressure distribution on the upper and lower walls of the slit outlet.

The pressure contours in Figure 10 reveal the mechanism of flow deflection affected by the temperature gradient. When the fluid flows from the slit to the wide region, there is normally a pressure drop near the slit outlet. The loss of pressure potential energy transforms into the promotion of kinetic energy, which speeds up the flow out of the slit and often forms a jet. Obviously, the pressure drop shows an almost symmetrical feature without heat input. Comparably, the pressure-drop area changes when the heat is applied. The introduction of heat reduces the viscosity and accelerates the fluid, which results in a non-uniform distribution of velocity and pressure in the flow field. The pressure drop on the heated side is larger than that on the unheated side. Due to the wide range of local pressure losses at both sides of outlet walls, the temperature gradient at the wall surface has little influence on the fluid flow. The defect is relevant to the temperature gradient between the two plates. Under the imbalance of pressure imposed on the fluid, the flow biases to the heated side. As the flow is fully developed at 4.12 s, a larger pressure gradient is produced between the heated and unheated walls, pushing more fluid into the heated side.

**Figure 11:**
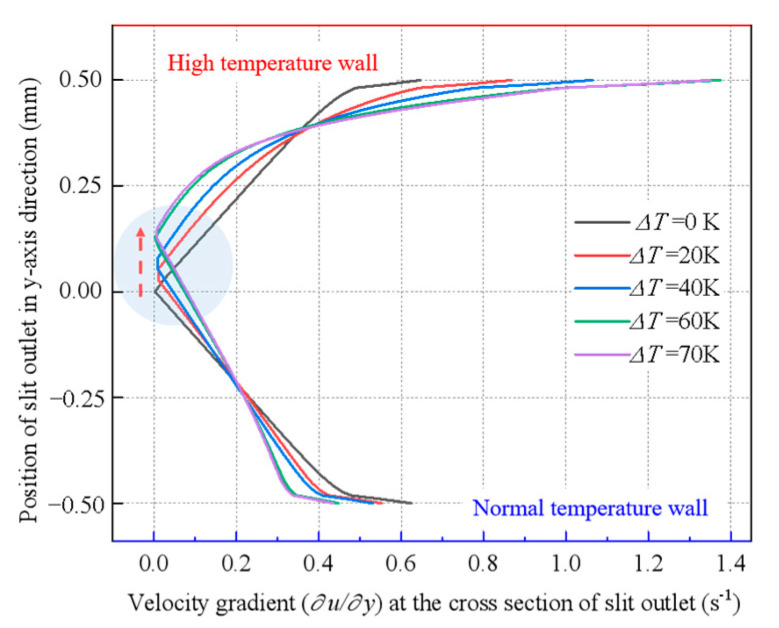
Velocity gradient diagram at the cross section of slit outlet at different temperature differences. The velocity gradient increases with the increase in temperature, and the turning point of the velocity gradient shifts towards the high temperature side.

To examine the flow variation under the pressure gradient, the velocity gradient in the *y*-axis direction at the cross-section of the slit outlet is investigated at 0.2 s. As shown in Figure 11, the velocity gradient distribution coincides well with the pressure contour feature. In the absence of a temperature difference, the velocity gradient distribution is axisymmetric, which means the position where the *∂p*/*∂x* is zero is at the center of the slit. With the increase in the temperature differences, the position of *∂p/∂x =* 0 moves off the axis and shifts up (referring to the shadow in Figure 11). Furthermore, the velocity gradient on the heated side enlarges tremendously in the flow boundary layer than that on the side at normal temperature, which presents a non-linear distribution. According to the theory of the boundary layer in laminar flow, in the flow direction, the resultant force of the total surface force of the fluid can be simplified as:

(10) $$F=\left(-\frac{\partial p}{\partial x}+\frac{\partial \tau_{xx}}{\partial x}+\frac{\partial \tau_{yx}}{\partial y}\right)dxdy$$

Here, *p* is the pressure and *τ* is the shear force. In the formula, the surface force can be divided into pressure and viscous force, indicating that a larger adverse pressure gradient and viscous resistance will accelerate the separation of the boundary layer. For steady laminar flow, (*∂p*/*∂x*) is usually a constant value [31]. It indicates that the flow characteristics are determined by the temperature-dependent viscosity. The variation of viscosity under the effect of a temperature gradient results in flow deflection in a slit. Specifically, the increase in the temperature of the fluid on the heating side wall reduces the viscosity of water, which results in lower viscosity resistance near the wall, allowing the fluid on the heating side to better adhere to the wall. Secondly, due to the deflection of the flow, the velocity of the fluid on the normal temperature side is low, resulting in less kinetic energy. With less kinetic energy but greater viscous resistance, the kinetic energy of the fluid on the normal temperature side will be consumed first, resulting in the separation of the boundary layer and deflection of the flow towards the heating side.

### 3.5. Switching Mechanism of Flow Attachment Locked by Temperature

**Figure 12:**
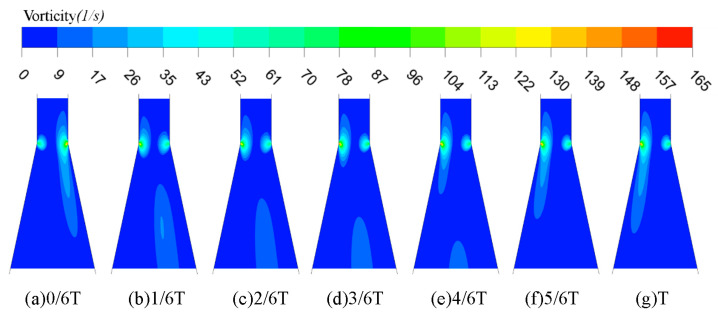
Development of vorticity with time in process of bistable flow switching (T represents a switching period, and 0/6 T is the switching heating wall moment). The maximum vorticity area on the right side gradually shifted to the left.

The heat input not only induces the deflection of the slit flow but also locks the flow to the heated side. The development of vorticity during the temperature-switching process is explored and depicted in Figure 12. Vorticity represents the curl of velocity, which expresses the rotation rate of fluid particles. Therefore, an increase in the vorticity means a local spin enhancement of fluid particles, which reveals the intensive disturbance induced by the temperature gradient in the fluid field. When heat is applied to the right side of the slit, as shown in Figure 12a, the vorticity near the heated wall is larger than that on the other side, and the largest vorticity appears in the boundary layer. The results agree with the feature of the velocity gradient. When the heat input is now switched to the other side, the thermal potential energy effect of the original heat receiving area is weakened (Figure 12b), and the change of vorticity first happens in the boundary layer and then spreads to the mainstream. Although the vorticity in the boundary layer varies quickly, it still takes a lot of time to transport the vorticity until the fluid field achieves the new balance. In the present study, it is assumed that the fluid density is kept at a constant value and that there is a no-slip boundary. The vorticity depends on the viscosity in accordance with the theory of vorticity dynamics. Consequently, variations in the viscosity of fluid are essential for reducing switching time.

### 3.6. Deflection Characteristics of Temperature-Driven with Disturbed Flow

The movement of fluids through a peristaltic pump, gear pump, and other power sources is called forced convection. And the input flow in these ways is usually a sine function of time. Therefore, this study explores the temperature control of variable velocity. In the slit, the sinusoidal function of velocity changing with time with a frequency of 0.5 Hz and disturbance rate of 50% is defined:

(11) $$u=0.02\sin\left(\pi t\right)+0.08$$

**Figure 13:**
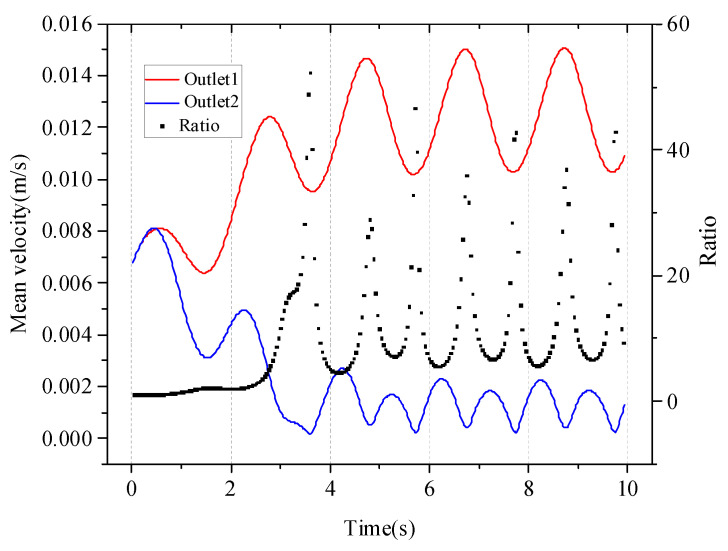
Variation of mean flow velocity at outlet1 (red line) and outlet2 (blue line) with time; the black scatter plot represents the ratio of outlet1 flow to outlet2 flow. All the times when the ratio is large correspond to the times when the flow velocity at outlet2 is low.

Equation (11) is taken as the first approximation. Based on this velocity change, the range of the Reynolds number of the slit segment can be obtained: 119–198. Figure 13 shows the specific velocity changes of the two outlets with time from the beginning of flow to 10 s. The red line represents the heating side outlet, and the blue line represents the normal temperature side outlet. In the figure, the flow ratio of the first 3 s of the thermal outlet to the normal temperature outlet gradually increases, indicating that the flow is still being controlled by temperature. After the flow is attached to the wall, both outlets will exhibit waveforms with a period of 2 s, and this frequency is consistent with the frequency that defines the speed switch. Among them, the points with a larger flow ratio are concentrated in the positions with the most and least flow at the heating side outlet. These positions have lower *∂u/∂t* values. Similarly, the position with a larger *∂u/∂t* has a smaller flow ratio. This indicates that flow perturbations can affect temperature control. However, even at the lowest position, the ratio can be 2.68 times higher than that without a temperature gradient, and it still has the ability to control the flow.

At the same time, the present study also explored the influence of variable Reynolds numbers on flow switching. The flow rate diagram for the switching period is similar to that of Figure 9, but the switching period is extended by 3.88 s. It shows that the unsteady velocity waveform also has a certain inhibitory effect on flow switching.

## 4. Conclusions

This research presents a two-dimensional numerical simulation to study the flow characteristics of bistable slit flow driven by temperature. The effect of temperature gradients on flow deflection and switching is investigated, and the mechanisms behind the phenomenon are explored. The main conclusions are drawn as follows:

(1) Temperature gradient can be a trigger to realize the control of bistable slit flow. The slit flow observably deflects and then is locked to the heated side under the impact of the temperature gradient. With the increase in the temperature gradient, the flow deflection angle becomes larger, which induces a jump in the flow mass ratio between the outlets. The ratio of slit length to slit width makes a difference in the flow deflection under the same temperature gradient, but the significance of the flow deflection is weaker after the ratio attains 10, which suggests there is an optimized structure parameter.
(2) The slit flow can be switched from one side to the other, driven by temperature. When the opening angle between the outlets is 24°, Reynolds number 167, and the slit temperature gradient is 40 K, the whole switching time costs 4.54 s and can be reduced by enlarging the temperature gradient.
(3) The mechanism of flow deflection and switching is investigated based on an analysis of the pressure distribution, the velocity gradient, and the vorticity development. It demonstrates that the heat input causes a pressure drop as well as an enhancement of the velocity gradient. The position where the shear stress is zero moves closer to the high-temperature wall with the amplified temperature difference. The development of vorticity in the switching process reveals that the variation of viscosity significantly determines the switching time.
(4) The ability of temperature to control the flow with disturbance is somewhat weakened, but for the fluid with a frequency of 0.5 Hz and a Reynolds number range of 119–198, temperature still has the ability to control.

The technique of temperature-driven switching of bistable flow enriches the active regulation of flow, which has great potential in the fields of mechanical engineering [32], bioengineering [33], medical engineering [34], and other relevant fields.

## Figures and Tables

**Table 1:** Average outflow velocity in the three types of grids.

	Grid Numbers	Outlet 1 Velocity (×10^−2^) (m/s)	Outlet 2 Velocity (×10^−4^) (m/s)
Coarse grids	9150	1.4542	2.3166
Medium grids	27,050	1.4507	2.4241
Fine grids	47,600	1.4508	2.4634
Deviation (%)		0.23%	5.96%

## Data Availability

Data are contained within the article.
